# Transfer learning-based modified inception model for the diagnosis of Alzheimer's disease

**DOI:** 10.3389/fncom.2022.1000435

**Published:** 2022-11-01

**Authors:** Sarang Sharma, Sheifali Gupta, Deepali Gupta, Sapna Juneja, Amena Mahmoud, Shaker El–Sappagh, Kyung-Sup Kwak

**Affiliations:** ^1^Department of Computer Science and Engineering, Chitkara University Institute of Engineering and Technology, Chandigarh, Punjab, India; ^2^Department of Computer Science, KIET Group of Institutions, Ghaziabad, India; ^3^Department of Computer Science, Kafrelsheikh University, Kafr el-Sheikh, Egypt; ^4^Faculty of Computer Science and Engineering, Galala University, Suez, Egypt; ^5^Information Systems Department, Faculty of Computers and Artificial Intelligence, Benha University, Banha, Egypt; ^6^Department of Information and Communication Engineering, Inha University, Incheon, South Korea

**Keywords:** feature visualization, modified inception, classification, confusion matrix, Alzheimer's disease

## Abstract

Alzheimer's disease (AD) is a neurodegenerative ailment, which gradually deteriorates memory and weakens the cognitive functions and capacities of the body, such as recall and logic. To diagnose this disease, CT, MRI, PET, etc. are used. However, these methods are time-consuming and sometimes yield inaccurate results. Thus, deep learning models are utilized, which are less time-consuming and yield results with better accuracy, and could be used with ease. This article proposes a transfer learning-based modified inception model with pre-processing methods of normalization and data addition. The proposed model achieved an accuracy of 94.92 and a sensitivity of 94.94. It is concluded from the results that the proposed model performs better than other state-of-the-art models. For training purposes, a Kaggle dataset was used comprising 6,200 images, with 896 mild demented (M.D) images, 64 moderate demented (Mod.D) images, and 3,200 non-demented (N.D) images, and 1,966 veritably mild demented (V.M.D) images. These models could be employed for developing clinically useful results that are suitable to descry announcements in MRI images.

## Introduction

Alzheimer's disease (AD) is a neurological condition that damages the neurons and slowly deteriorates memory and hampers basic cognitive functions and abilities. This disease is detected by monitoring changes in the brain, which eventually result in neuron loss and their connections. According to the WHO, around 50 million people suffer from dementia, and nearly 10 million new cases of AD are reported every year. Ultimately, AD destroys the part of the brain that controls breathing and heart monitoring, eventually leading to fatality. AD involves three stages: very mild, mild, and moderate (Feng et al., 2020; Rallabandi et al., 2020). However, an individual affected by AD begins to show symptoms at the moderate stage. This disease affects the communication between neurons.

In the mild stage, progressive deterioration eventually hinders independence, with patients unable to perform most of the common activities of daily living. Speech difficulties become evident due to an inability to recall vocabulary, which leads to frequent incorrect word substitutions. Reading and writing skills are also progressively lost. Complex motor sequences become less coordinated with time as the disease progresses, so the risk of falling increases. During this phase, memory problems worsen, and the patients may fail to recognize even close relatives. Long-term memory, which was previously intact, becomes impaired. Moreover, old age alone does not cause AD, several health, environmental, and lifestyle factors also contribute to AD (Ebrahimi-Ghahnavieh et al., 2019; Talo et al., 2019; Nakagawa et al., 2020), including heart disease, lack of social engagement, and sleep (Hon and Khan, 2017; Aderghal et al., 2018; Islam and Zhang, 2018).

This study utilizes a novel modified inception-based model that classifies AD into four sub-categories: V.M.D, M.D, Mod.D, and N.D. The model was run on a large MRI dataset (Jha et al., 2017). The following research points can be inferred from the study:

1. A modified inception v3 model was implemented to classify AD into four classes.
2. This model was modified by adding six convolutional layers, four dense block layers, two dropout layers, one flattening layer, and one layer with an activation function.
3. Image enhancement and augmentation processes were utilized to expand the image quantities in the dataset.

The proposed model was implemented using an Adam optimizer and 1,000 epochs.

## Background literature

Most of the research work has applied the binary classification of AD (Jha et al., 2017; Aderghal et al., 2018; Ebrahimi-Ghahnavieh et al., 2019; Rallabandi et al., 2020) and a smaller dataset to design their proposed model, which may not be adaptable. The researchers (Hon and Khan, 2017; Talo et al., 2019; Feng et al., 2020; Nakagawa et al., 2020; Rallabandi et al., 2020) working on a large dataset have implemented two output-based classifications (Ali et al., 2016) or classifications of binary inputs (Bin. C) (Kang et al., 2021), which resulted in only marginal accuracy (Li et al., 2021). Table 1 compares the existing state-of-the-art models.

**Table 1:** Literature survey of existing models.

**Ref**.	**Journal**	**Techniques**	**Aim**	**Challenges of the approach**
Rallabandi et al. (2020)	Informatics in Medicine Unlocked	Non-Linear SVM with 2D CNN	To develop an automated technique to classify normal, early and late mild AD individuals	Dataset contained 1,167 brain MRI images. It utilized Bin.C and showed 75% accuracy
Feng et al. (2020)	International Journal of Neural Systems	3D CNN-SVM	To distinguish Mod. D and M.D individuals from N.D individuals, for improving value-related care of Mod. D individuals in medical facilities	Dataset contained 3,127, 3T T1-weighted MRI brain images. It utilized classification of three inputs and showed 88.9% accuracy. It also aims to focus on regressing Mod. D individuals to healthy individuals predict Mod. D progression and improve in diagnosis of AD in future
Nakagawa et al. (2020)	Brain Communications	Cox, DeepHit	To diagnose conversion time from normal individual to AD individual by using deep survival analysis model	Dataset contained 2,142, T1-weighted images. It utilized classification of three inputs and showed 92.3% accuracy. It aims to diagnose the group of M.D individuals that would convert to AD in future
Ebrahimi-Ghahnavieh et al. (2019)	IAICT	GoogleNet, AlexNet, VGGNet16, VGGNet19, SqueezeNet, ResNet18, ResNet50, ResNet101, inceptionv3	To detect AD on MRI scans using D.L techniques	Dataset contained 177 images. It utilized Bin.C and showed 84.38% accuracy. To comprise PET scans in the system to examine several aspects of AD
Talo et al. (2019)	Computerized Medical Imaging and Graphics	AlexNet, VGGNet16, ResNet18, ResNet34, ResNet50	To diagnose MRI images into N.D and Mod.D	Dataset contained 1,074; T2-weighted MRI images and it utilized classification of multiple inputs and showed 95.59% accuracy
Islam and Zhang (2018)	Brain Informatics	inceptionV4, ResNet, ADNet	To diagnose AD by utilizing Deep-CNN ensemble	Dataset contained 416; T1-weighted sMRI scans and it utilized classification of multiple inputs and showed 93.18% accuracy. To predict AD from proposed model other brain diseases
Aderghal et al. (2018)	CBMS	Data Augmentation, CNN	To classify AD analysis by using Cross-Modal Transfer Learning	Dataset contained 416; sMRI image scans and it utilized Bin.C and showed 82.1% accuracy. To utilize a longitudinal dataset and implement cross modal method based on ROI spatial optimization
Hon and Khan (2017)	BIBM	CNN, Transfer Learning	To classify AD by using cross modal transfer learning algorithms	Dataset contained 6,400 brain images and yielded 92.3% accuracy while utilizing binary classification
Jha et al. (2017)	Journal of Healthcare Engineering	DTCWT, PCA, (FNN)	To develop a CAD system to early diagnose AD individulas	Dataset contained 416; T1- weighted image scans and it implemented binary classification and yielded an accuracy of 90.06%. To test 3D-DTCWT, wavelet packet analysis, utilize ICA, LDA and PCA
Ali et al. (2016)	International Journal of Computer Applications	VGG16, inceptionV4	To classify AD by utilizing transfer learning algorithms in pre-trained models	Dataset contained 416; MRI AD and MCI image scans were utilized and utilized Bin.C and showed 74.12% accuracy by scratch and 92.3% by transfer learning algorithms
Kang et al. (2021)	CBM	2D-CNN, VGG16	To classify AD by using ensemble based CNN	Dataset contained 798; T1-weighted image scans and it utilized Bin.C and showed 90.36% accuracy. To distinguish AD from MCI images by using 2D-CNN
Li et al. (2021)	BMRI	SVM, CNN	To distinguish MCI from AD by using SVM classifier with linear kernel	Dataset contained 1,167; T1-weighted image scans and it utilized Bin.C and showed 69.37% accuracy. To distinguish AD from MCI images by using SVM-CNN
Venugopalan et al. (2021)	SR	SVM, k-NN, CNN	To distinguish MCI from AD by using SVM and k-NN	Dataset contained 1,311; T1 and T2 weighted image scans and utilized Bin.C and showed 75% accuracy. To distinguish AD from MCI images by using SVN-CNN, KNN

## Materials and methods

**Figure 1:**
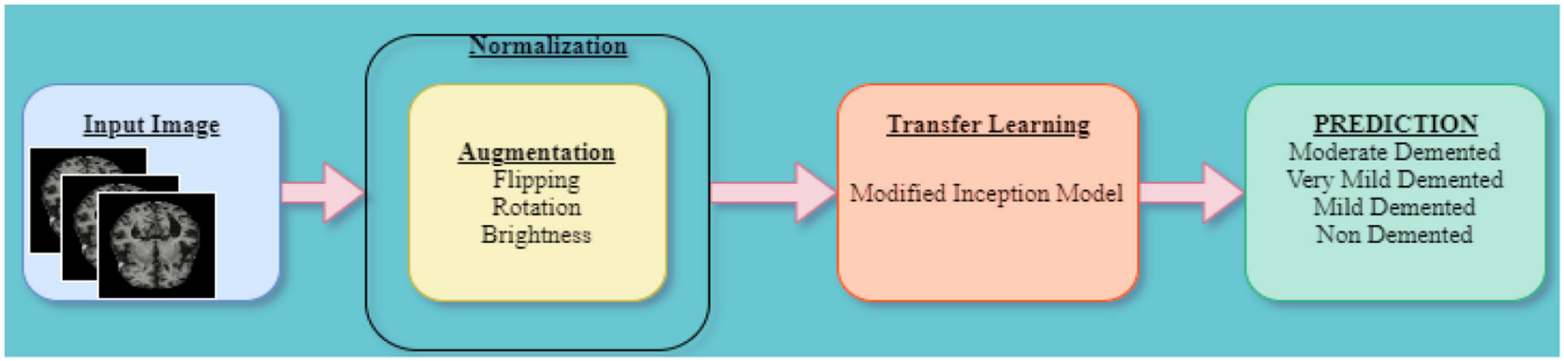
Proposed Alzheimer's disease detection model.

This model classifies AD into ND, VMD, MD, and Mod D classes (Sarraf and Tofighi, 2016). The proposed model utilizes the Kaggle dataset containing 6,200 AD images. The model involves augmentation of data (Zhao et al., 2017) and extraction of features using a modified inception model (Fan et al., 2008), as shown in Figure 1. The model is executed using the Keras package in Python with Tensorflow, which is used at the backend of Intel(R) Core (TM) i5-6400 CPU 2.70 GHz processors and 12GB RAM.

### Input dataset

**Figure 2:**
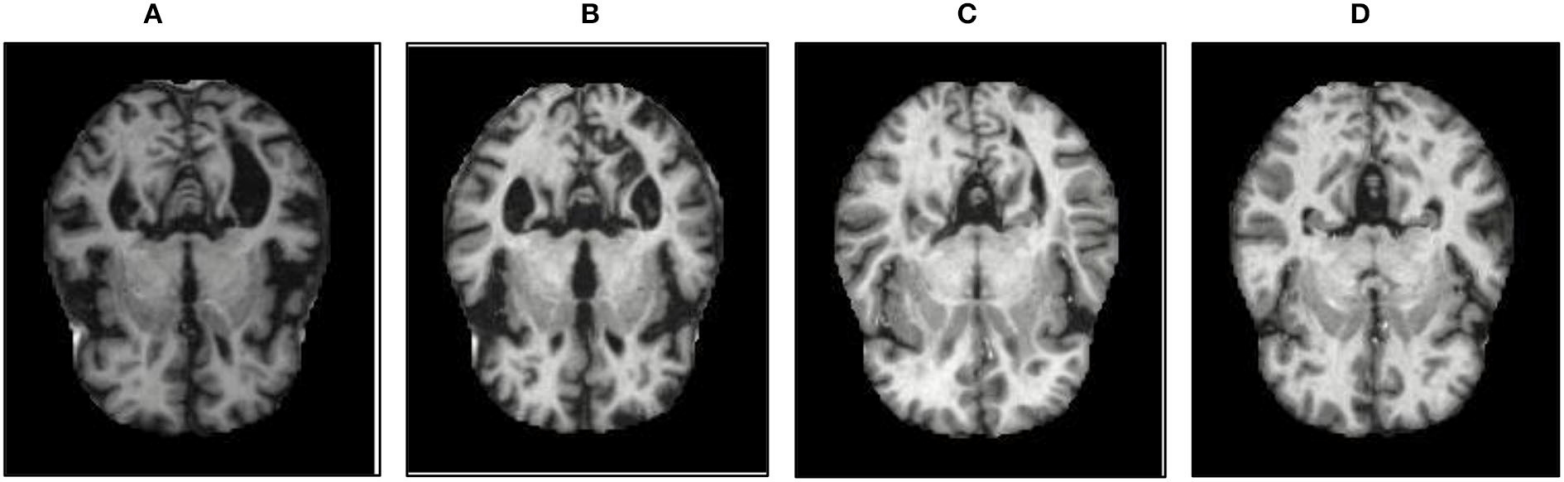
Alzheimer's disease: **(A)** M.D, **(B)** Mod.D, **(C)** N.D, and **(D)** V.M.D.

The database used in this study consists of a total of 6,200 AD images that are retrieved from the Kaggle database. It comprises grayscale images of 896 MD, 64 Mod D, 3,200 ND, and 1,966 VMD images, with a dimension of (208 × 176 × 3) pixels. The dataset for evaluation is divided in such a way that 80% of the image samples are utilized for training the model and the remaining 20% are utilized for testing the model (Filipovych et al., 2011). Figure 2 shows the database of MRI images. Table 2 shows the publicly available AD dataset.

**Table 2:** Publicly available Alzheimer's disease dataset.

**Dataset**	**Classes**	**Class name**	**Class images**	**Total images**
OASIS	4	N.D	292	416
		V.M.D	24	
		M.D	28	
		Mod.D	72	
ADNI	3	ND	159	469
		MD	157	
		Mod.D	153	
Harvard Medical School	4	Mod.D	378	1,680
Kaggle	4	M.D	896	6,126
		Mod.D	64	
		V.M.D	1,966	
		N.D	3,200	

Table 3 shows the dataset description in which the number of training images, testing images, and validation images are given for AD classes. The total number of images in the dataset is 6,200, of which 179 are MD, 12 are Mod.D, 640 are ND, and 448 are VMD images. The complete dataset is divided into training and validation (Misra et al., 2009; Moradi et al., 2015).

**Table 3:** Alzheimer's dataset description.

**S.No**.	**Alzheimer**	**Training**	**Validating**	**Before**	**After**	**Training**	**Validating**
		**images**	**images**	**augmentation**	**augmentation**	**images**	**images**
1	M.D	717	179	896	2,688	2,150	538
2	Mod.D	52	12	64	640	512	128
3	N.D	2,560	640	3,200	3,500	2,800	700
4	V.M.D	1,518	448	1,966	3,932	3,145	787

### Normalization

Data normalization preserves the numerical stability of the modified inception model (Serra et al., 2016; Rathore et al., 2017). The MRI images have values ranging from 0 to 255. By utilizing the normalization technique, the images in the proposed model are trained faster (Rashid et al., 2022).

### Augmentation

To enhance usefulness, a dataset with the maximum number of samples is required, but numerous site, privacy, and data restrictions often accompany while acquiring the dataset.

**Figure 3:**
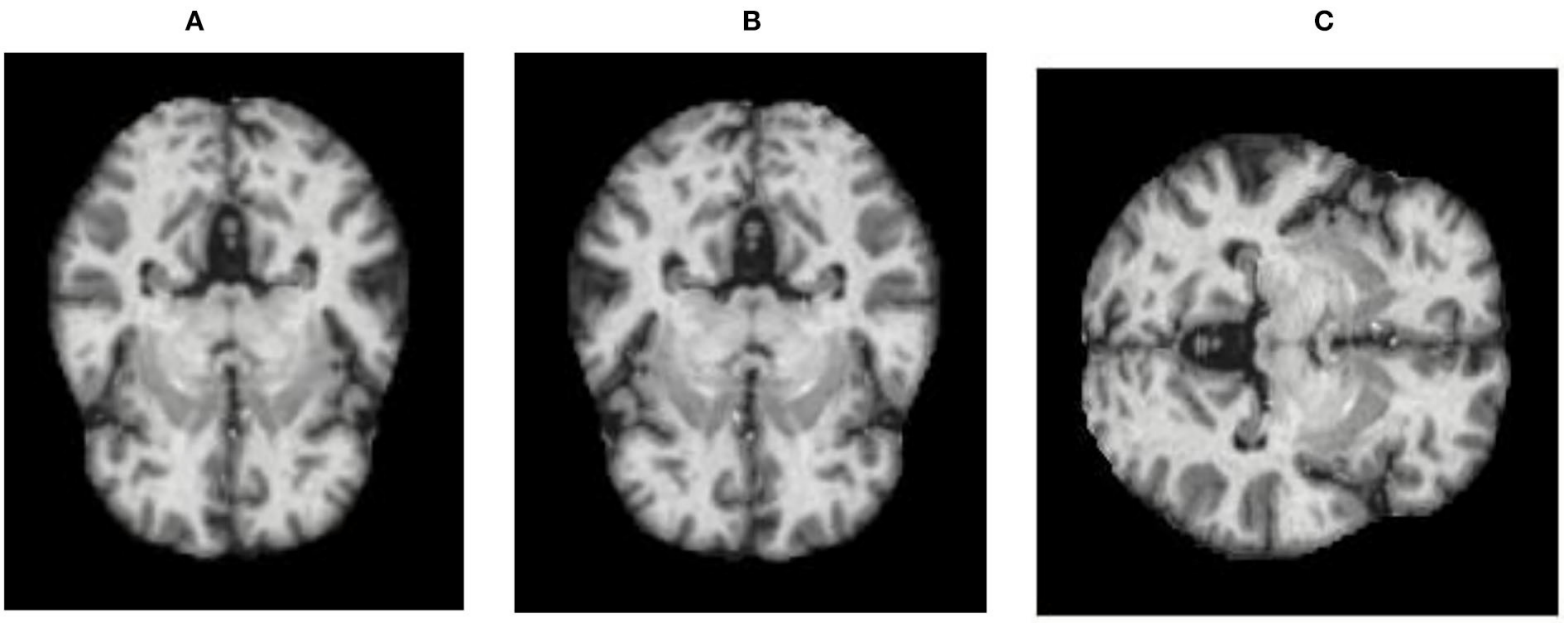
FL applied to the dataset: **(A)** original, **(B)** HF, and **(C)** VF.

**Figure 4:**
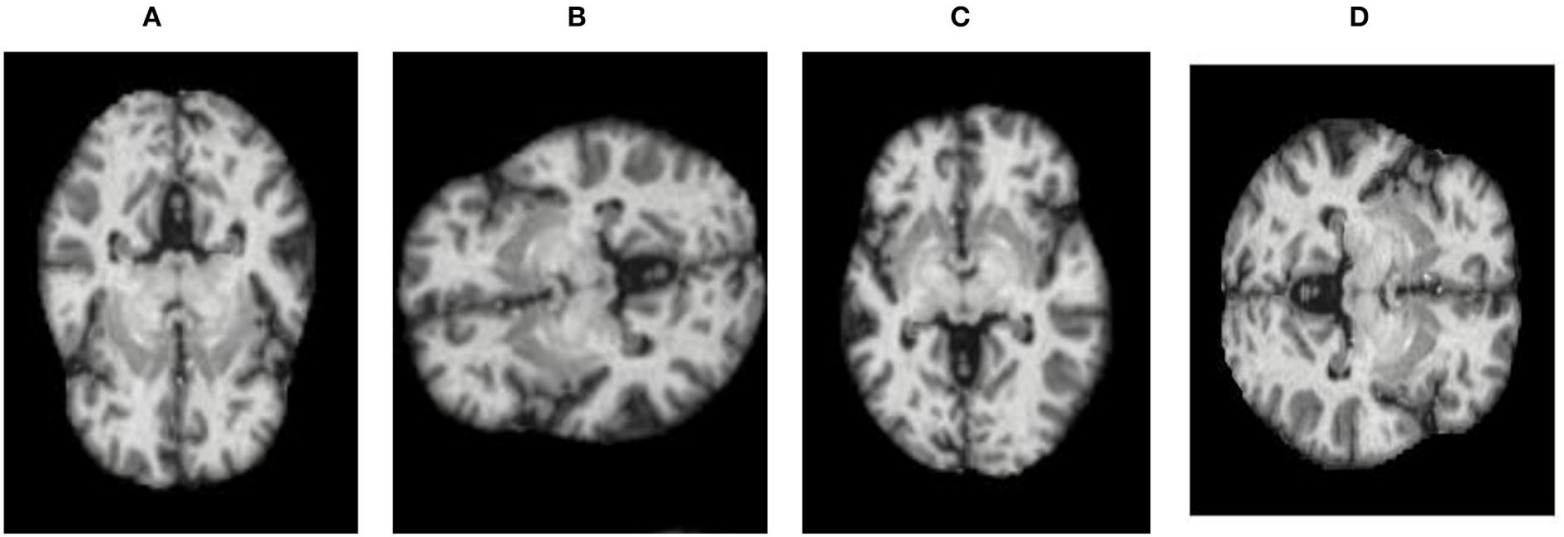
Ro applied to the dataset: **(A)** original, **(B)** 90 degrees anticlockwise, **(C)** 180 degrees anticlockwise, and **(D)** 270 degrees anticlockwise.

**Figure 5:**
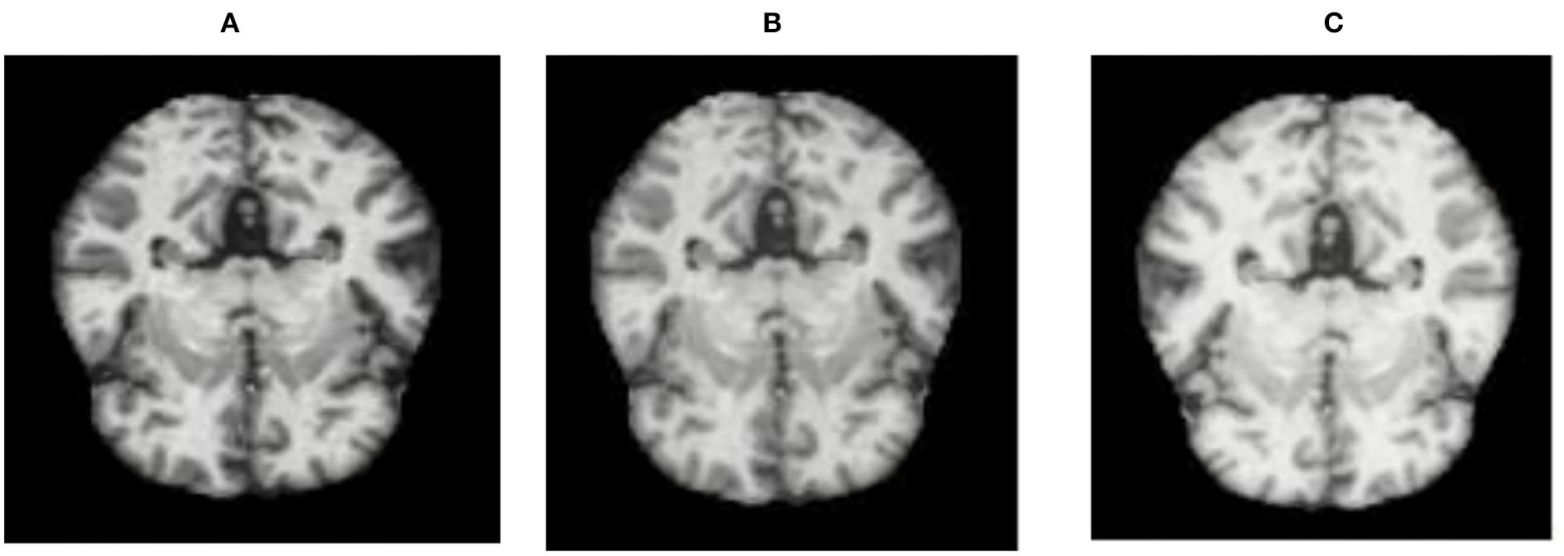
Bss applied to the dataset: **(A)** original image, **(B)** with Bss factor 0.3, and **(C)** with Bss factor 0.7.

Therefore, to overcome these issues, augmentation of data is performed, which increases the original data quantity. Augmentation includes flipping (FL), rotation (Ro), and brightness (Bss). Both vertical (VF) and horizontal flipping (HF) techniques (Dhankhar et al., 2021; Juneja A. et al., 2021; Juneja S. et al., 2021) are shown in Figure 3. The Ro technique, as shown in Figure 4, is implemented in an anticlockwise direction by an angle of 90 degrees each. Bss, as shown in Figure 5, is also applied to the image dataset by taking brightness factor values as 0.3 and 0.7.

Table 3 exhibits the number of images before and after data augmentation. Furthermore, a disproportion in the number of images was found in every class. To improve this disproportion (Sharma et al., 2022c), augmentation of data was performed, as mentioned before. After their execution, the samples increased from 6,200 to 10,760 images, which represent the updated images. This is applied only to the training images. Before augmentation, the training images of MD, Mod D, ND, and VMD were 896, 64, 3,200, and 1,966, respectively. After the augmentation, the total number of training images became 10,760, which represents the total number of images of training and validation data after augmentation.

### Feature extraction using the modified inception model

**Figure 6:**
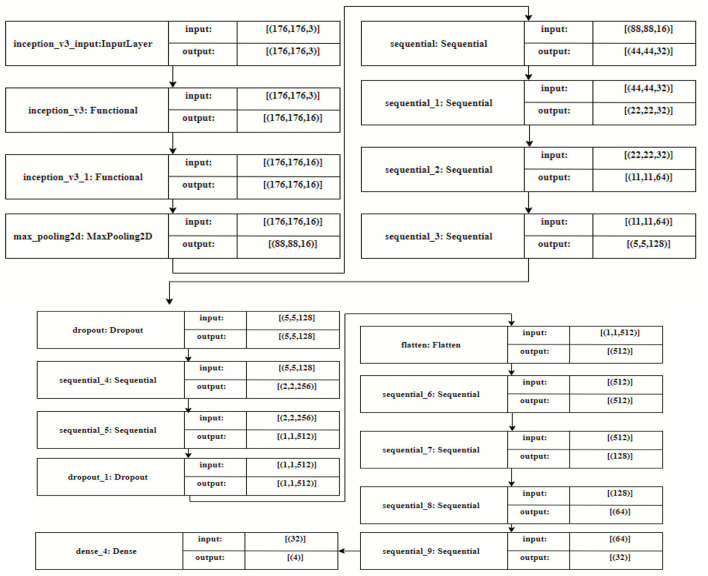
Modified inception architecture.

In the proposed model, input images with a dimension of 208 ^*^ 176 pixels were applied, as shown in Figure 6. The modified inception architecture consisted of 12 blocks. In the first and second blocks, two inception layers of size 3 and one max pooling layer of size 2 with 32, 64, and 128 filters, respectively; in the third and fourth blocks, two convolution layers with 32 filters; and in the fifth and sixth blocks with one convolution layer with 256 and 128 filters, respectively, were applied. These layers were followed by a dropout layer with 128 filters. The seventh and eighth layers consisted of 256 and 512 filters, respectively, followed by another dropout layer with 128 filters. Then, the flattened layer was connected with 512 filters, and the ninth, 10th, 11th, and 12th dense layers consisted of 512, 128, 64, and 32 filters, respectively. At last, the fully connected layers were implemented, and the classified output was obtained.

Table 4 exhibits the filter visualization image of every convolution layer. The single kernel or filter for each convolutional layer is mentioned.

**Table 4:** Filter visualization for every convolution layers.

**First convolution layer**					
inception_v3_input	inception_v3	inception_v3_1	max_pooling2d	sequential	sequential_1
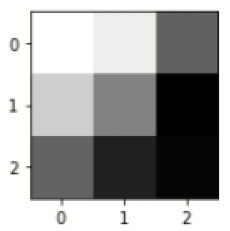	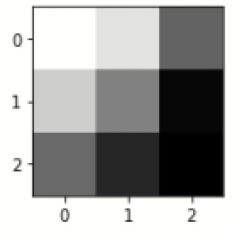	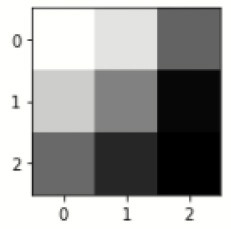	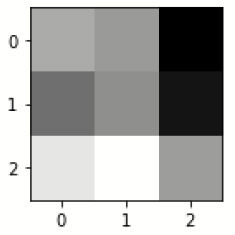	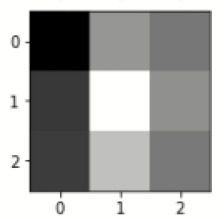	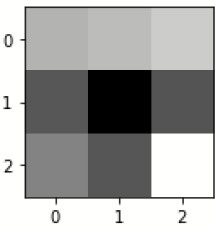
**Last convolution layer**					
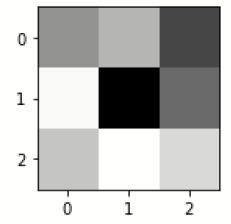	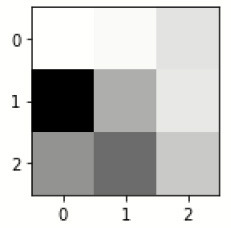	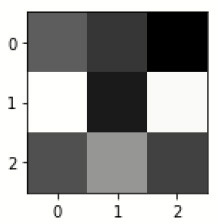	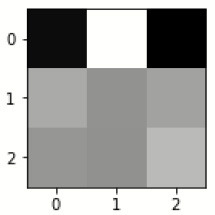	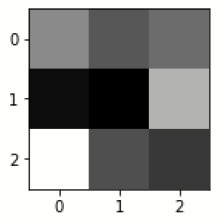	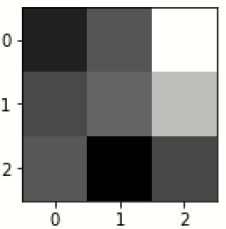
**Filter for first convolution layer**					
sequential_2	sequential_3	dropout	sequential_4	sequential_5	dropout_1
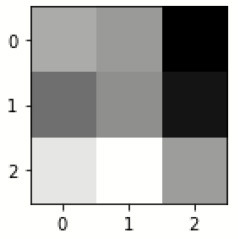	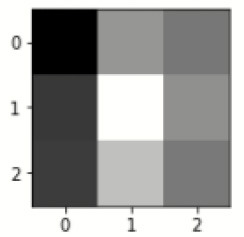	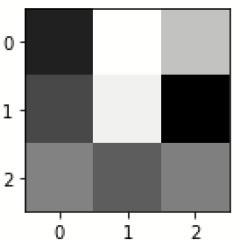	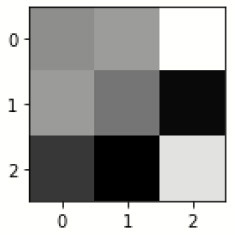	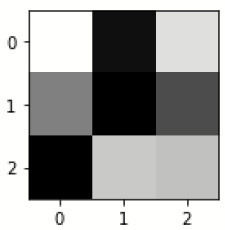	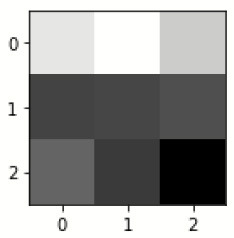
**Filter for last convolution layer**					
sequential_2	sequential_3	dropout	sequential_4	sequential_5	dropout_1
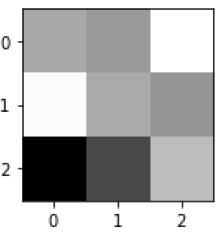	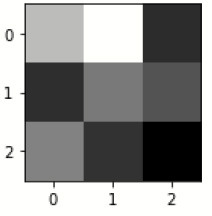	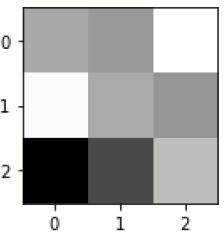	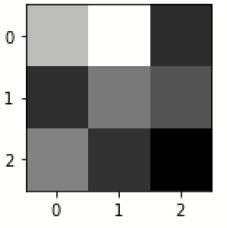	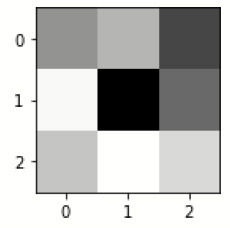	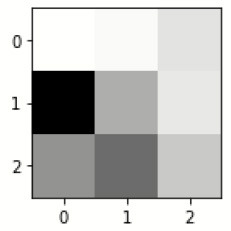

The images are filtered with the help of kernels, as given in Table 5 as it displays the feature-visualized images of each convolutional layer (Chugh et al., 2022; Dhiman et al., 2022). It displays the first and last feature-visualized images for every convolutional layer (Sharma et al., 2022a,b).

**Table 5:** Images after each dense block.

**Filter for first convolution layer**					
inception_v3_input	inception_v3	inception_v3_1	max_pooling2d	sequential	sequential_1
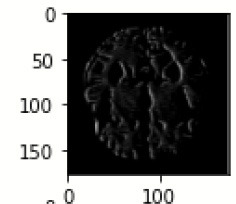	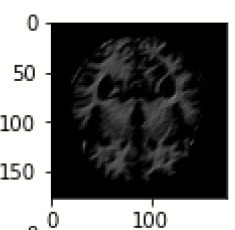	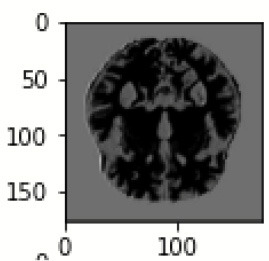	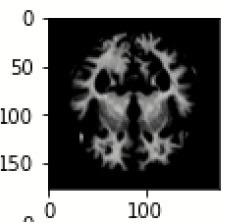	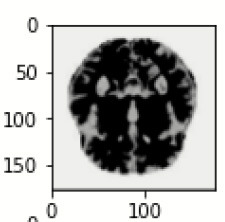	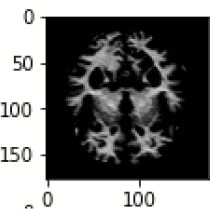
**Filter for last convolution layer**					
inception_v3_input	inception_v3	inception_v3_1	max_pooling2d	sequential	sequential_1
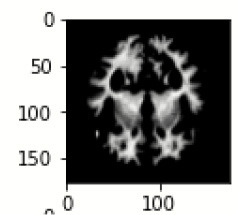	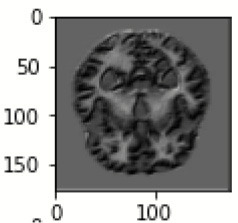	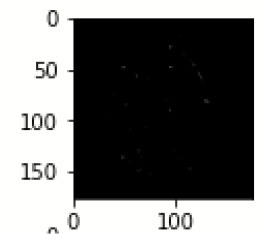	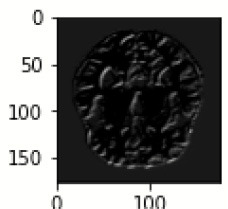	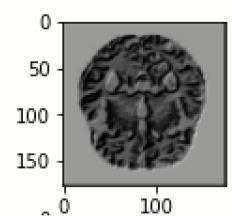	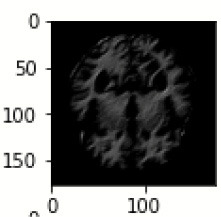
**Filter for first convolution layer**					
sequential_2	sequential_3	dropout	sequential_4	sequential_5	dropout_1
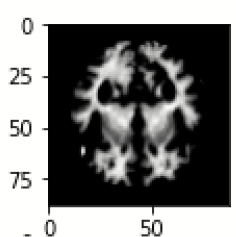	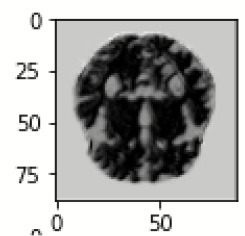	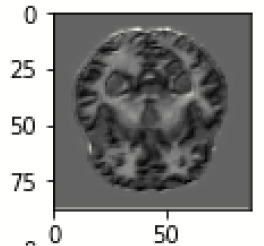	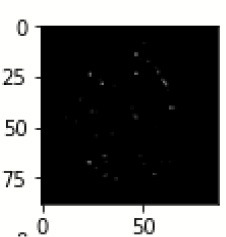	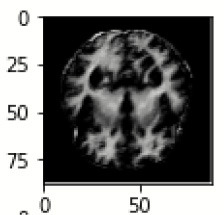	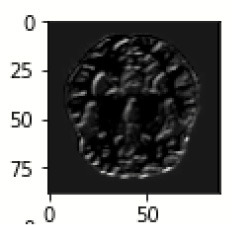
**Filter for last convolution layer**					
sequential_2	sequential_3	dropout	sequential_4	sequential_5	dropout_1
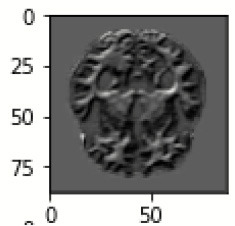	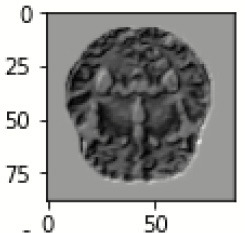	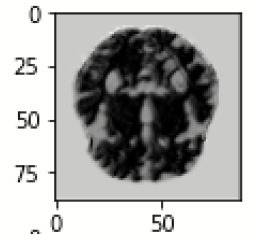	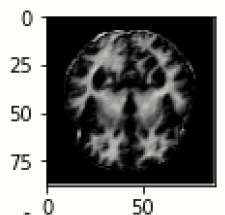	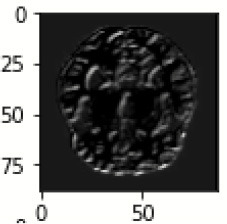	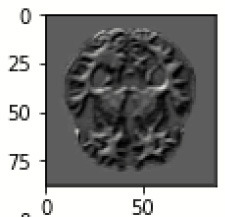

## Results

Various tuning parameters were applied to AD images, like optimizer, batch size (BS), and epochs, which modified neural network features and thus minimized the losses. The Adam optimizer was used in this model. BS specifies managed images in a single iteration. BS 32 was utilized in these models. A total of 1,000 epochs were used in these models. The Adam optimizer was used for training the deep learning algorithms as it includes both functionalities of AdaGrad and RMSProp optimizers. Large BS results in heavy computational processes during deep learning model training, whereas small BS results in a faster computational process. Hence, there is always a trade-off between large and small BS. The number of epochs should be more so that error can be minimized during model training; however, a large number of epochs increase the computational time. In this study, the simulation of the proposed model is carried out using 1,000 epochs. Table 8 shows the name of hypertuning parameters and their values.

### Confusion matrix

**Figure 7:**
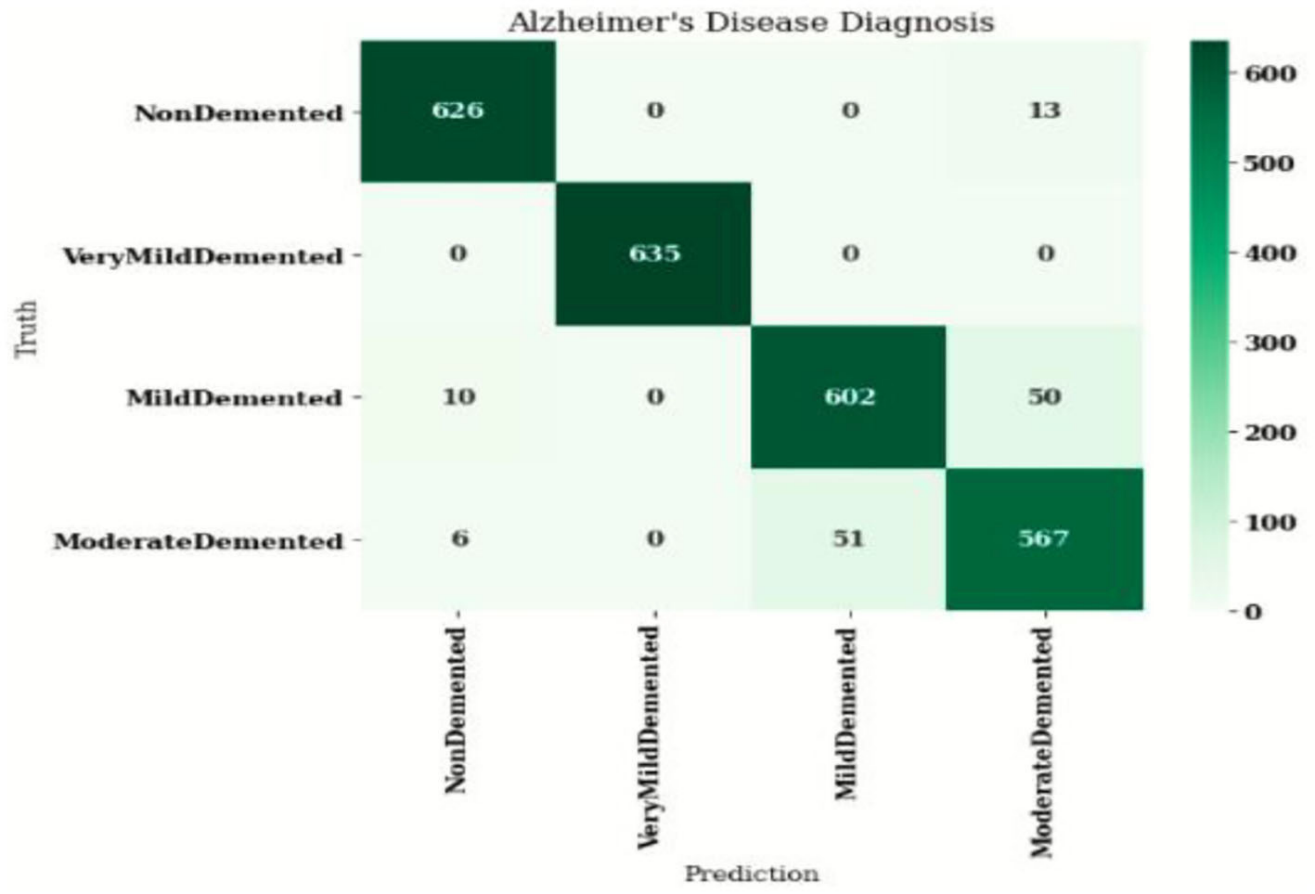
Confusion matrix for the modified inception model.

Figure 7 shows the confusion matrix, which represents classification predictions. The accuracy of the entire model is 94.92%. The confusion matrix parameters are converted by classification report. These confusion matrix parameters are given as follows:

- Accuracy (Acc) is the ratio of true predictions to observed predictions, as in Equation 1:
  (1) $$\begin{array}{l} Accuracy=\frac{TP+TN}{TP+TN+FP+FN} \end{array}$$
- Precision (Prec) is the ratio of correct positive predictions to the total positive predictions, which can be given by Equation 2:
  (2) $$\begin{array}{l} Precision=\frac{TP}{TP+FP} \end{array}$$
- Specificity (Spec) is the ratio of correct negative predictions to the total negatives, which can be given by Equation 3:
  (3) $$\begin{array}{l} Specificity=\frac{TN}{TN+FP} \end{array}$$
- Sensitivity (Sens) is the ratio of correct positive predictions to the total positives, which is given by Equation 4:
  (4) $$\begin{array}{l} Sensitivity=\frac{TP}{TP+FN}. \end{array}$$

Figure 7 displays the confusion matrix for the modified inception model. The accuracy value of the proposed model is 94.92%.

**Figure 8:**
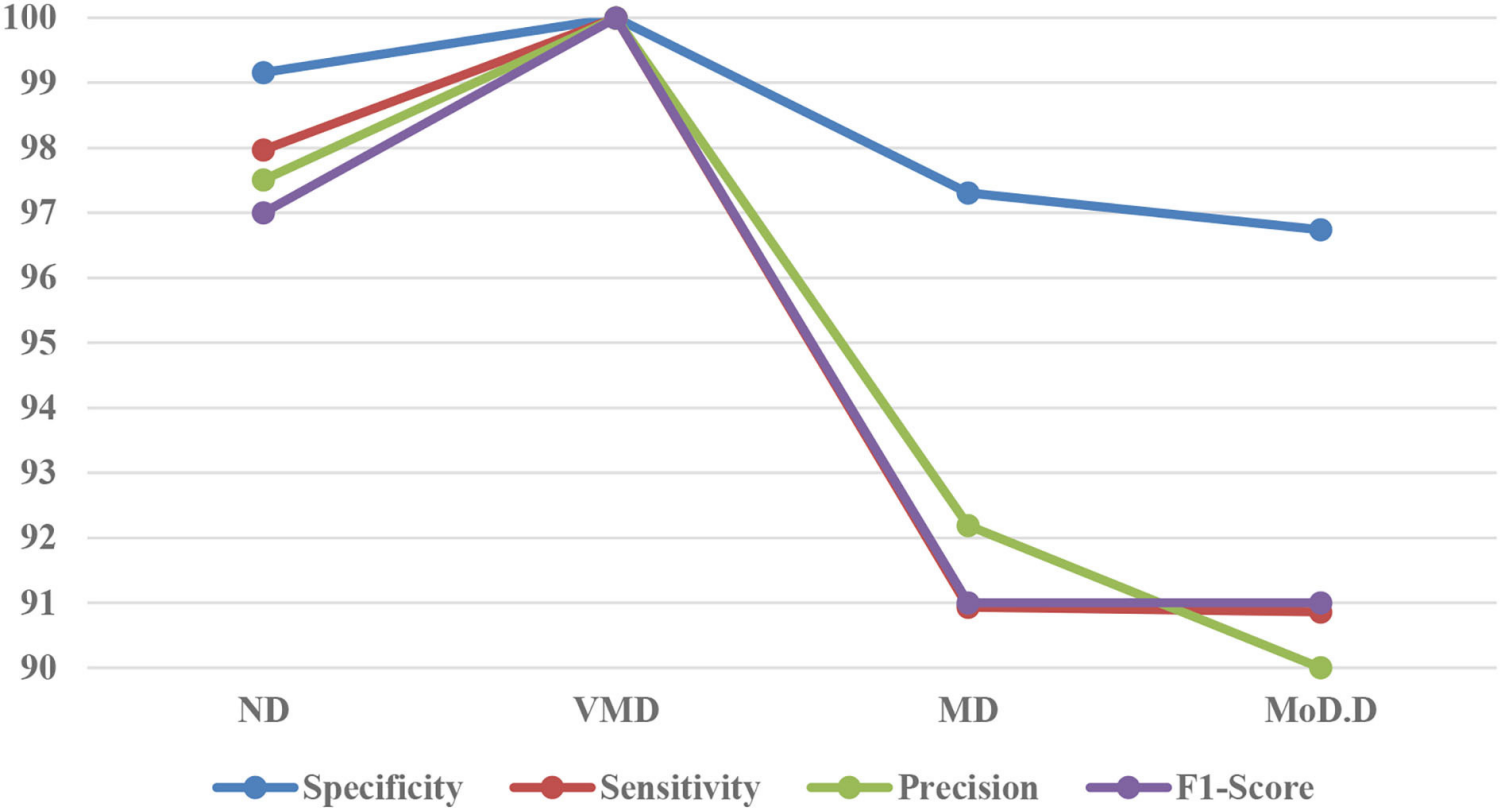
Graph of confusion matrix parameters with F1-Score.

Figure 8 exhibits the precision, sensitivity, and specificity values for all AD classes for a batch size of 32 with the Adam optimizer. In Figure 8, V.M.D exhibits a maximum precision of 100%, followed by ND, with a maximum precision of 97.51%. Also, V.M.D exhibits a sensitivity of 100%, followed by N.D, with a sensitivity of 97.97%. Furthermore, V.M.D displays a specificity of 100%, followed by N.D with a specificity of 99.16%. The average Prec, Sens, and Spec of a batch size model of 32 with Adam, Adadelta, and SGD optimizers are exhibited in Tables 6–8, respectively.

**Table 6:** Confusion matrix constituents of the modified inception model with the Adam optimizer.

**AD type**	**Precision**	**Sensitivity**	**Specificity**	**F1-score**
N.D	97.51	97.97	99.16	97
V.M.D	100	100	100	100
M.D	92.19	90.93	97.31	91
Mod.D	90	90.86	96.74	91
Avg. precision	94.93	–	–	–
Avg. sensitivity	–	94.94	–	–
Avg. specificity	–	–	98.3	–
				94.75

**Table 7:** Confusion matrix constituents of the modified inception model with the Adadelta optimizer.

**AD Type**	**Precision**	**Sensitivity**	**Specificity**
N.D	94.23	96.13	97.52
V.M.D	100	100	100
M.D	91.7	88.57	95.21
Mod.D	84.43	86.61	92.5
Avg. precision	92.59	–	–
Avg. sensitivity	–	92.83	–
Avg. specificity	–	–	96.31

**Table 8:** Confusion matrix constituents of the modified inception model with the SGD optimizer.

**AD type**	**Precision**	**Sensitivity**	**Specificity**
N.D	97.62	98.6	99.2
V.M.D	100	100	100
M.D	94.5	92.27	98.33
Mod.D	91.7	92.5	96.2
Avg. precision	95.9	–	–
Avg. sensitivity	–	95.84	–
Avg. specificity	–	–	98.43

A comparison of all the optimizers is shown in Table 9, where the SGD optimizer showed better average precision, average sensitivity, and average specificity than both Adam and Adadelta optimizers.

**Table 9:** Confusion matrix constituents of the modified inception model with the SGD optimizer.

**Optimizers**	**Avg. precision**	**Avg. sensitivity**	**Avg. specificity**
SGD	95.9	95.84	98.43
Adam	94.93	94.94	98.3
Adadelta	92.59	92.83	96.31

Similarly, the average Prec, Sens, and Spec of a batch size of 64 in the inception model with Adam, Adadelta, and SGD optimizers are exhibited in Tables 10–12, respectively. By adding Gaussian NB to the last layer of the inception model of a batch size of 32 with the Adam optimizer, the results denote a significant increase in performance parameters, as shown in Table 13.

**Table 10:** Confusion matrix constituents of the modified inception model with the Adam optimizer.

**AD type**	**Precision**	**Sensitivity**	**Specificity**
N.D	91.6	92.5	96.3
V.M.D	100	100	100
M.D	90.11	88.7	95.17
Mod.D	85.7	86.2	92.8
Avg. precision	91.85	–	–
Avg. sensitivity	–	91.86	–
Avg. specificity	–	–	96.06
Avg. F1-score	–	–	–

**Table 11:** Confusion matrix constituents of the modified inception model with the Adadelta optimizer.

**AD type**	**Precision**	**Sensitivity**	**Specificity**
N.D	89.38	91.13	93.71
V.M.D	100	100	100
M.D	87.32	88.57	94.93
Mod.D	84.43	85.14	91.17
Avg. precision	90.28	–	–
Avg. sensitivity	–	91.21	–
Avg. specificity	–	–	94.95

**Table 12:** Confusion matrix constituents of the modified inception model with the SGD optimizer.

**AD type**	**Precision**	**Sensitivity**	**Specificity**
N.D	95.13	96.5	97.51
V.M.D	100	100	100
M.D	93.91	90.2	95.8
Mod.D	89.18	89.82	92.13
Avg. precision	94.55	–	–
Avg. sensitivity	–	94.13	–
Avg. specificity	–	–	96.36

**Table 13:** Confusion matrix constituents of the modified inception model with the Gaussian NB classifier.

**AD type**	**Precision**	**Sensitivity**	**Specificity**
N.D	97.8	98.21	99.38
V.M.D	100	100	100
M.D	96.25	91.97	98.5
Mod.D	93.12	93.8	96.41
Avg. precision	96.79	–	–
Avg. sensitivity	–	95.9	–
Avg. specificity	–	–	98.57

## Discussion

**Figure 9:**
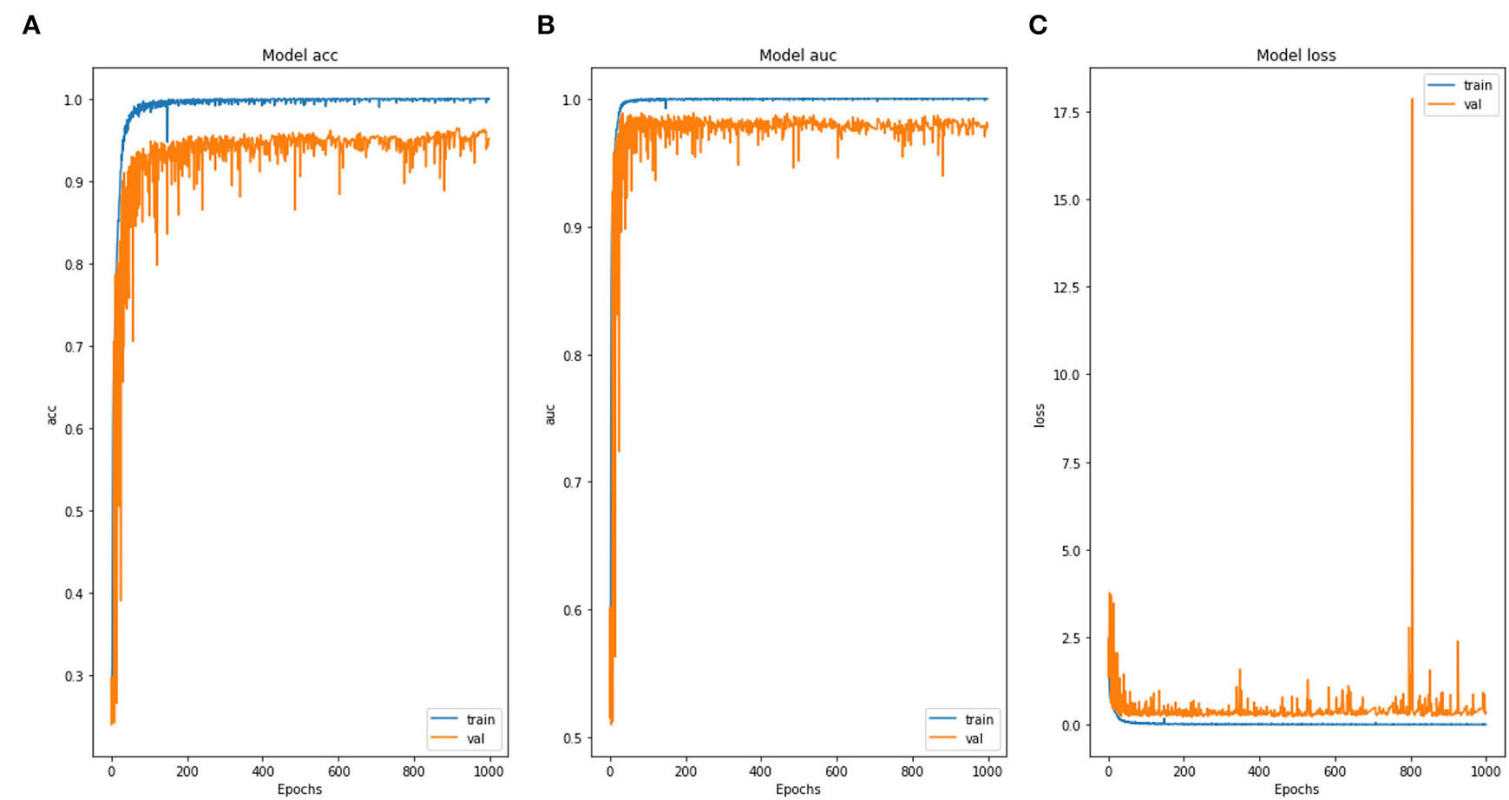
Modified inception model graphs containing **(A)** model accuracy, **(B)** model AUC, and **(C)** model loss.

For the training of the proposed model, the Adam optimizer was utilized. Confusion matrix parameters and training performance parameters for the model are shown in Figure 8. From Figure 9, it can be inferred that this model obtained the comparatively highest parametric values with a Prec of 94.93%, a Sens of 94.94%, a Spec of 98.3%, and a yielded Acc of 94.92%. Model accuracy was used for evaluating classification models, and model loss was used for optimizing parameter values. Figure 9A displays the graphs of training accuracy and validation accuracy for the modified inception model, from which it can be inferred that training accuracy was better than validation accuracy for all the epochs. Figure 9B shows the graphs of the training area under the curve (AUC) and validation area under the curve, from which it can be deduced that the AUC for training data was 1, whereas the AUC was <1 for validation data. Figure 9C shows the graphs of training loss and validation loss for the modified inception model, from which it can be inferred that validation loss is high only at the 800th epoch; otherwise, its value is <0.5.

From Table 14, it can be deduced that at the 1,000th epoch, the training Acc value is maximum at BS 32, that is, 95.11%, whereas training loss is minimum, that is, 0.3483. Furthermore, at the 1,000th epoch, the training Acc value is maximum at BS 64, that is, 93.57%, whereas training loss is minimum, that is, 0.3442.

**Table 14:** Training performance of the modified inception model with the Adam optimizer.

**Epoch**	**Train**	**Train**	**Validation**	**Val**
**value**	**loss**	**accuracy**	**loss**	**accuracy (%)**
**For batch size 32**				
200	0.0182	0.9964	0.2894	0.9413
400	0.0111	0.9969	0.3890	0.9482
600	0.0067	0.9987	0.3421	0.9502
800	0.0022	0.9996	0.7948	0.9419
1,000	0.0017	0.9998	0.3483	0.9517
**For batch size 64**				
200	0.0212	0.9836	0.3577	0.9223
400	0.0152	0.9897	0.3463	0.9267
600	0.0082	0.9923	0.3861	0.9109
800	0.0065	0.9946	0.3458	0.9291
1,000	0.0023	0.9971	0.3442	0.9357

### Performance evaluation with previous implementations

Results obtained from the model are displayed in Table 10 which shows that the model achieved better parametric values than previous models due to several pre-processing methods. However, some studies have utilized comparatively larger image datasets to validate their models (Hon and Khan, 2017; Talo et al., 2019; Feng et al., 2020; Nakagawa et al., 2020; Rallabandi et al., 2020). Furthermore, Bin.C was achieved in most studies; previous studies have also performed tertiary or multiclass classification (Talo et al., 2019; Feng et al., 2020; Nakagawa et al., 2020). Table 15 displays the comparison between the proposed and existing models.

**Table 15:** Comparison with previous implementations.

**Authors**	**Database**	**Images**	**Techniques**	**Weighted Acc**
Rallabandi et al. (2020)	ADNI	1,167	SVM with D.L	75%
Feng et al. (2020)	ADNI	3,127	2D-CNN with D.L	82.57%
Nakagawa et al. (2020)	ADNI	2,142	Cox model with D.L	92%
Ebrahimi-Ghahnavieh et al. (2019)	ADNI	177	DenseNet-201 ResNet50	84.38% 81.25%
Talo et al. (2019)	Harvard Medical School	1,074	VGG16	92.49%
Islam and Zhang (2018)	OASIS	416	ResNet50	93.18%
Aderghal et al. (2018)	OASIS	416	Cross-Modal Transfer Learning	83.57%
Hon and Khan (2017)	Kaggle	6,400	VGG16	92.3%
Jha et al. (2017)	OASIS	416	DTCWT and PCA with FNN	90.06%
Ali et al. (2016)	OASIS	416	VGG16	92.3
Kang et al. (2021)	ADNI	798	2D-CNN, VGG16	90.36%
Li et al. (2021)	ADNI	1,167	SVM, CNN	69.37%
Venugopalan et al. (2021)	ADNI	1,311	SVM, k-NN, CNN	75%
Proposed methodology	Kaggle	6,400	Transfer Learning Based Modified inception Model	94.92%

## Conclusion

In this study, the effectiveness of the proposed model for the discovery of announcements has been completely estimated. The dataset for the announcement was acquired from Kaggle by one of the authors (Sarvesh Dubey). The results were attained after the training and analysis of these models. Furthermore, by duly working the optimizer and images, these results demonstrated the effectiveness of the proposed models. Acc and Sens of 94.92 and 94.94 independently were achieved with the proposed model with the Adam optimizer. The study models performed better in both training and testing, with similar results.

A possible limitation would be to guarantee reproducibility; however, this issue could be solved by using a large brain MRI dataset. A transfer learning-based approach places the convolution information into machine learning parts and the AD images into deep learning parts before adding both the results of the processes. This study helps for a more accurate opinion for the development of D.L model. Different transfer learning-based models and optimization processes would also be employed to further enhance the effectiveness of the proposed model. Medical image analysis is one of the grueling tasks with useful computational methods on the scale of imaging operations.

## Data availability statement

The raw data supporting the conclusions of this article will be made available by the authors, without undue reservation.

## Author contributions

SS, SG, DG, and SJ contributed to conception and design of the study. AM organized the database and performed the statistical analysis. SE–S wrote the first draft of the manuscript. K-SK wrote sections of the manuscript. All authors contributed to manuscript revision, read, and approved the submitted version.

## Funding

This work was supported by the National Research Foundation of Korea through a grant funded by the Korean government (Ministry of Science and ICT)-NRF-2020R1A2B5B02002478.

## Conflict of interest

The authors declare that the research was conducted in the absence of any commercial or financial relationships that could be construed as a potential conflict of interest.

## Publisher's note

All claims expressed in this article are solely those of the authors and do not necessarily represent those of their affiliated organizations, or those of the publisher, the editors and the reviewers. Any product that may be evaluated in this article, or claim that may be made by its manufacturer, is not guaranteed or endorsed by the publisher.
